# Comparative determination of HIV-1 co-receptor tropism by Enhanced Sensitivity Trofile, gp120 V3-loop RNA and DNA genotyping

**DOI:** 10.1186/1742-4690-7-56

**Published:** 2010-06-30

**Authors:** Mattia CF Prosperi, Laura Bracciale, Massimiliano Fabbiani, Simona Di Giambenedetto, Francesca Razzolini, Genny Meini, Manuela Colafigli, Angela Marzocchetti, Roberto Cauda, Maurizio Zazzi, Andrea De Luca

**Affiliations:** 1 Infectious Diseases Clinic, Catholic University of Sacred Heart, Rome, Italy; 2 Molecular Biology Department, University of Siena, Siena, Italy; 3 Infectious Diseases Unit, University Hospital of Siena, Siena, Italy

## Abstract

**Background:**

Trofile^® ^is the prospectively validated HIV-1 tropism assay. Its use is limited by high costs, long turn-around time, and inability to test patients with very low or undetectable viremia. We aimed at assessing the efficiency of population genotypic assays based on gp120 V3-loop sequencing for the determination of tropism in plasma viral RNA and in whole-blood viral DNA. Contemporary and follow-up plasma and whole-blood samples from patients undergoing tropism testing via the enhanced sensitivity Trofile^® ^(ESTA) were collected. Clinical and clonal geno2pheno_[coreceptor] _(G2P) models at 10% and at optimised 5.7% false positive rate cutoff were evaluated using viral DNA and RNA samples, compared against each other and ESTA, using Cohen's kappa, phylogenetic analysis, and area under the receiver operating characteristic (AUROC).

**Results:**

Both clinical and clonal G2P (with different false positive rates) showed good performances in predicting the ESTA outcome (for V3 RNA-based clinical G2P at 10% false positive rate AUROC = 0.83, sensitivity = 90%, specificity = 75%). The rate of agreement between DNA- and RNA-based clinical G2P was fair (kappa = 0.74, p < 0.0001), and DNA-based clinical G2P accurately predicted the plasma ESTA (AUROC = 0.86). Significant differences in the viral populations were detected when comparing inter/intra patient diversity of viral DNA with RNA sequences.

**Conclusions:**

Plasma HIV RNA or whole-blood HIV DNA V3-loop sequencing interpreted with clinical G2P is cheap and can be a good surrogate for ESTA. Although there may be differences among viral RNA and DNA populations in the same host, DNA-based G2P may be used as an indication of viral tropism in patients with undetectable plasma viremia.

## Background

Maraviroc (MVC) is the first CCR5 antagonist approved for the treatment of HIV-1 infection [1] following the demonstration of its virological efficacy in treatment-experienced patients [2,3]. There is reasonable expectation that MVC or other CCR5-antagonists can be even better administered to treatment-naïve patients due to a higher prevalence of CCR5-tropic (R5) HIV-1 in this population as compared to more advanced patients [4]. Due to the lack of virologic activity against CXCR4-tropic (X4) virus [5], the administration of MVC is subject to the verification of an R5 virus population in the candidate patient. The enhanced sensitivity Trofile^**® **^assay (ESTA) is the current gold standard phenotypic method for the determination of co-receptor tropism for the replicating viral population (i.e. plasma RNA), although other in-house or commercial tests are available, some of which use peripheral blood mononuclear cell (PBMC DNA) [6,7]. The drawbacks of any phenotypic test include high costs, long turn-around time, and reduced efficiency in patients with low viremia. For this reason, there is a demand for a fast and cheap HIV-1 tropism assay to fully exploit CCR5 antagonists as a treatment option in clinical routine [8,9].

Given that most of the determinants of viral co-receptor tropism are based on polymorphisms of the third hypervariable region (V3) of the gp120, an alternative to the phenotypic approach is the usage of machine learning tools based on viral genotypic information. So called in-silico or virtual phenotype models may be indeed convenient for clinical practice due to the reduction of costs and turn-around time. During the recent years, several prediction models have been studied, from the first simple rule based on the polymorphisms at V3 codons 11 and 25, to the position specific scoring matrices (PSSM), neural networks, support vector machines, random forests and logistic models [10-20]. Some of these studies identified additional factors possibly impacting viral tropism, such as viral subtype and CD4 cell counts [14,16,20]. Comparisons among genotypic and phenotypic tests have been carried out. The genotypic geno2pheno_[coreceptor] _system [16] was compared with the first generation Trofile^**® **^and the TRT phenotypic assays [21], showing 86.5% and 79.7% concordance, respectively. A study comparing the predictive performance of geno2pheno_[coreceptor]_, PSSM [12] and other methods against the first-generation Trofile^**® **^assay, concluded that current default implementations of co-receptor prediction algorithms were inadequate for predicting CXCR4 co-receptor usage in clinical samples, due to inability to detect low-level X4 virus [22]. Another study found the concordance among genotype-based predictors and first-generation Trofile^**® **^being as high as 91% [23]. Variable performance of in-silico models was shown when considering non-B HIV-1 variants [24-26].

Concerning the clinical validation of phenotypic assays, another recent work focused on the performance of the Trofile^**® **^test in predicting the virological response to a short-term maraviroc exposure in HIV-infected patients [27]. Concomitantly, a few attempts to unveil mutational patterns associated to selection by CCR5 antagonists or resistance have been carried out [28,29], but the frequency and rate at which maraviroc resistance mutations emerge are not yet known.

The most awaited information is how in-silico tropism prediction models predict virological response to CCR5-antagonists, particularly when genotypic and phenotypic results disagree. In fact, although the ESTA should detect lower amounts of X4 virus compared to bulk genotyping, the X4 level threshold compromising in vivo CCR5-antagonist activity in vivo is currently unknown. In addition, ESTA cannot be performed at low or undetectable viral load (VL), while HIV-1 DNA genotyping can be easily performed in such cases; and this information, if adequately validated, might be easily employed for guiding treatment switches to CCR5 antagonists in virologically suppressed patients, due to toxicity or simplification issues. Finally, genotyping can also be used to detect HIV-1 mutations selected by MVC and possibly decreasing its effectiveness. Our study aimed at evaluating the accuracy of HIV-1 co-receptor tropism prediction by viral RNA and DNA genotyping, as well as the selection of V3 mutants in MVC-failing individuals.

## Methods

### Patients

Contemporary plasma and whole-blood samples were prospectively collected from HIV-infected patients followed up at a single centre of the Infectious Diseases Clinic of Catholic University of Sacred Heart in Rome, Italy, all failing antiretroviral treatment and potentially candidates for treatment with a CCR5-antagonists, in the period between November 2007 and July 2009 (n = 55). Some of these patients underwent tropism testing via the ESTA (n = 51). Follow-up plasma and whole-blood samples from these patients were also collected, regardless of MVC treatment.

### Viral amplification and sequencing

Plasma RNA and whole-blood DNA were obtained from citrated blood by spin column extraction (Qiagen, Hilden, Germany). Plasma underwent 1-hour centrifugation at 23,000 x g at 4°C to concentrate virus prior to extraction. Whole blood was used without pre-processing steps. A 419-bp region encompassing the HIV-1 gp120 V3 domain was amplified by (RT)-PCR and sequenced in both strands by infrared-labelled primers on a Licor IR2 system. Plasma RNA was reverse transcribed with primer P151 (5'-CTACTTTATATTTATATAATTCAYTTCTC-3', coordinates 7661-7689 in the reference HXB2 genome). The reverse transcription reaction was run for 30 minutes at 37°C and included 10 μL of RNA extract, 50 mM Tris-HCl (pH 8.3 at 25°C), 75 mM KCl, 10 mM DTT, 3 mM MgCl_2_, 200μM each dNTP, 200 U ImProm II RT (Promega), 20 U RNasin (Promega) and 5 pmol primer P151. The cDNA obtained (one third of the reverse transcription mixture) or blood DNA (1 μg) extracted from whole-blood was amplified by a nested PCR protocol using primer P150 (5'-AATGTCAGCACAGTACAATGYACACAT-3', 6945-6971) and P151 in the outer amplification step and primer LR33 (5'-CAGTACAATGTACACATGGAAT-3', 6955-6976) and LR34 (5'-GAAAAATTCCCCTCCACAATT-3', 7353-7373) in the inner amplification step. Both outer and inner PCR mixtures contained 50 mM Tris-HCl (pH 9.0 at 25°C), 50 mM KCl, 2 mM MgCl_2_, 200 μM each dNTP, 1.25 U GoTaq polymerase (Promega) and 8 pmol each primer. The cycling profile was 20 seconds at 52°C, 40 seconds at 72°C and 30 seconds at 94°C for both steps but the number of cycles was 25 in the outer PCR and 32 in the inner PCR. The final product was sequenced by the IRD700-labelled sense primer IR25 (5'-GCTGTTAAATGGCAGTCTAGCAGAA-3', 7011-7035) and the IRD800-labelled antisense primer IR77 (5'-GAAAAATTCTCCTCYACAATTRA-3', 7351-7373) using the DYEnamic Direct Cycle Sequencing kit with 7-deaza-dGTP (GE Healthcare). Sequence contigs were assembled by the DNASTAR SeqMan II version 5.07 module. Only one PCR product per sample was subjected to standard population sequencing, expected to allow detection of minority species contributing at least 20% of the whole virus quasispecies.

### Sequence analysis: tropism prediction and its evaluation

The geno2pheno_[coreceptor] _in-silico genotypic tropism prediction system was employed, using both clinical (requiring VL, nadir CD4 and CD8 count) and clonal interpretations at 10% false positive rate (FPR) [17], and by considering a clonal interpretation at the optimised cutoff of 5.75% FPR, based on analysis of clinical data from MOTIVATE and MERIT studies [30,31]. Concordances among geno2pheno predictors and ESTA were assessed by Cohen's kappa statistic [32]. The predictive ability of the systems was evaluated by receiver operating characteristic (ROC) analysis [33,34].

The distance between nucleotide V3 sequences was calculated by the maximum composite likelihood [35], over a multiple alignment obtained via MUSCLE [36]. Phylogenetic analysis was performed using the MEGA 4 software [37], estimating tree and branch support with a bootstrapped neighbour-joining method.

Analysis of selection of env mutations by MVC exposure was performed by considering patients with a RNA sequence before (the closest to the start date) and after (considering the latest after at least one month of therapy) MVC initiation. Amino acid mutations were extracted by local pairwise alignments against the HXB2 reference sequence. Fisher's exact test on frequency counts of individual mutations and pre- post-MVC strata was executed; obtained p-values were also corrected with Benjamini-Hochberg procedure.

The R mathematical programming suite was used to perform all statistical analyses [38].

## Results

### Study population and samples

We processed 178 samples (99 plasma RNA and 79 whole-blood DNA) and successfully amplified 155 samples [78 (78.8%) plasma RNA and 77 (97.5%) whole-blood DNA] from 55 patients. By stratifying for contemporary VL, the rate of successful sequencing from RNA was 40%, 88.2%, and 96.2% at < = 50 cp/ml, 51-500 cp/ml, and >500 cp/ml respectively. The rate of successful sequencing from DNA was 95.1%, 100%, and 100% at < = 50 cp/ml, 50-500 cp/ml, and >500 cp/ml, respectively.

Fifty-one of the 55 patients had their baseline plasma tested by ESTA, and 28 were subsequently administered MVC. Patients' baseline characteristics are summarized in Table 1.

**Table 1:** Patients' characteristics at the time of ESTA

No. of patients with available ESTA result		51		
ESTA results, n (%)		CCR5-tropic	31 (61%)	
		
		
		Dual/Mixed-tropic	12 (23%)	
		
		CXCR4-tropic	1 (2%)	
		
		Not Reportable	7 (14%)	

*Descriptive*		*total*	*CCR5-tropic*	*Dual/Mixed/CXCR4-tropic*

Female gender, n (%)		16 (31%)	12 (39%)	4 (31%)

Mean age (SD)		45 (9)	46 (8)	40 (12)

Median time from HIV-1 diagnosis, years (IQR)		17 (14-19)	16 (13-18)	18 (17-22)

Italian nationality, n (%)		48 (94%)	29 (93%)	12 (92%)

Risk Factor, n (%)	Heterosexual	15 (29%)	11 (35%)	3 (23%)
	
	Homosexual/Bisexual	17 (33%)	11 (35%)	4 (31%)
	
	IDU	14 (27%)	7 (23%)	3 (23%)
	
	Other/Unknown	5 (10%)	2 (6%)	3 (23%)

CDC stage, n (%)	A	16 (32%)	11 (35%)	3 (25%)
	
	B	10 (20%)	9 (29%)	1 (8%)
	
	C	24 (48%)	11 (35%)	8 (67%)

History of mono-dual NRTI therapy, n (%)		37 (72%)	24 (77%)	10 (77%)

Median duration of ART exposure, years (IQR)		14 (11-16)	13 (11-16)	13 (11-15)

No. of drug switches (any change or interruption) experienced (IQR)		13 (9-16)	13 (9-15)	11 (7-15)

Patients previously exposed to drug class, n (%)	NRTI	50 (98%)	31 (100%)	13 (100%)
	
	NNRTI	44 (86%)	29 (93%)	10 (77%)
	
	Unboosted PI	49 (96%)	30 (97%)	13 (100%)
	
	Boosted PI	35 (69%)	22 (71%)	8 (61%)

CD4 count, median cells/mm3 (IQR)		334 (182-535)	387 (214-464)	211 (198-518)
HIV-1 RNA load, median log10 cp/ml (IQR)		3.66 (2.55-4.30)	3.9 (3.6-4.1)	4.2 (4.0-4.8)
CD4 count at nadir, median cells/mm3 (IQR)		64 (18-191)	138 (31-200)	14 (6-41)
HIV-1 RNA load at zenith, median Log10 cp/ml (IQR)		5.3 (4.6-5.7)	5.5 (4.8-5.7)	5.4 (4.5-5.6)

In a cross sectional crude analysis, using dichotomized ESTA results (R5-tropic versus X4- plus dual/mixed-tropic), we did not find any significant association with contemporary patients characteristics, except for the time from HIV-1 diagnosis [median (IQR) 16 years (13-18) for R5, and 18 years (17-22) for X4 or dual/mixed-tropic isolates p = 0.007], and nadir CD4 count [median (IQR) 138 cells/mm3 (31-200) for R5 and 14 cells/mm3 (6-41) for X4 or dual/mixed-tropic isolates p = 0.05].

### Comparison between geno2pheno_[coreceptor] _classification of plasma HIV-1 RNA, DNA V3 sequences and ESTA tropism results

**Figure 1:**
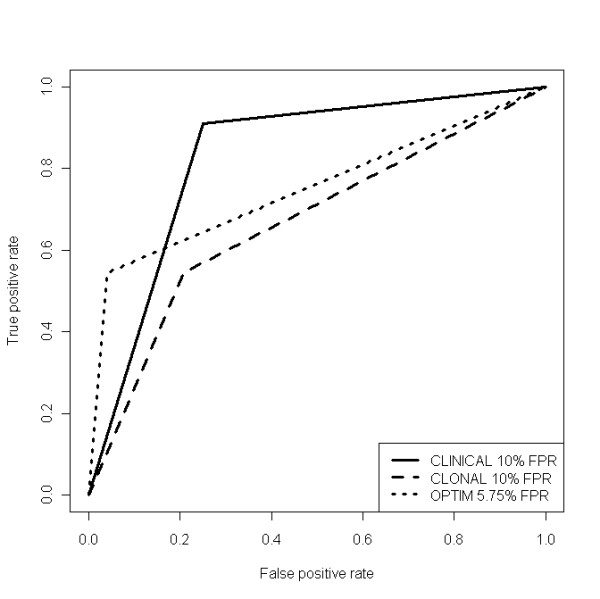
**Area under the ROC curves (AUROC) comparing predictions from RNA samples using geno2pheno clinical (AUROC = 0.83) at 10% FPR, geno2pheno clonal (AUROC = 0.67) at 10% FPR, and clonal at 5.75% FPR cutoff (AUROC = 0.75) interpretation modes versus the ESTA result (n = 35)**.

We first compared the prediction performance of geno2pheno versus the ESTA result. For this analysis, we excluded samples not reportable by ESTA. To match geno2pheno categories, the dual/mixed virus classification by ESTA was pooled with the X4 virus classification. Figure 1 depicts ROC plots with the performance of two RNA-based geno2pheno models predicting the ESTA X4 or dual/mixed tropism. Using nucleotide V3 loop sequences obtained from RNA samples contemporary to ESTA testing (n = 35), the resulting area under the ROC curve (AUROC) was 0.83 for geno2pheno clinical interpretation, 0.67 for geno2pheno clonal interpretation at 10% FPR, and 0.75 using the optimised 5.75% FPR cutoff. When comparing differences in AUROC with respect to the clinical interpretation reference, neither the clonal interpretation at 10% FPR nor that at 5.75% FPR cutoff exhibited a statistically significant lower area (p = 0.17 and p = 0.48, respectively).

**Figure 2:**
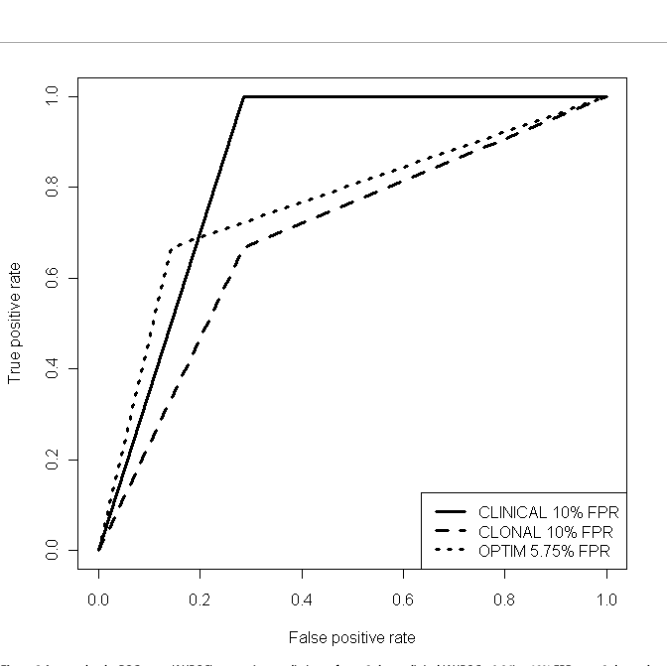
**Area under the ROC curve (AUROC) comparing predictions of geno2pheno clinical (AUROC = 0.86) at 10% FPR, geno2pheno clonal (AUROC = 0.69) at 10% FPR, and clonal at 5.75% FPR cutoff (AUROC = 0.76) interpretation modes versus the ESTA result, using sequences obtained from contemporary DNA samples (n = 17)**.

As a second performance test, we obtained geno2pheno tropism predictions for V3 sequences obtained from DNA samples (n = 17, with 16 sequences from patients with contemporary RNA genotyping). The resulting prediction of ESTA X4-D/M tropism showed an AUROC of 0.86 using geno2pheno clinical interpretation, 0.69 using the geno2pheno clonal interpretation at 10% FPR, and 0.76 using the optimised 5.75% FPR cutoff (Figure 2). We did not find significant differences in AUROC when comparing either the clonal interpretation (p = 0.34) at 10% FPR or that at 5.75% FPR cutoff (p = 0.58) against the clinical geno2pheno interpretation at 10% FPR.

Table 2 shows accuracy, sensitivity and specificity of the clinical, and clonal interpretation at 10% or 5.75% FPR cutoff of RNA/DNA geno2pheno interpretation modes in predicting ESTA X4-D/M-tropism.

**Table 2:** Performance evaluation of ESTA X4-dual/mixed tropism prediction using geno2pheno clinical and clonal at 10% FPR, or the clonal at the optimised 5.75% FPR interpretation of contemporary viral gp120 V3 DNA or RNA genotyping

Viral sample	geno2pheno interpretation mode (FPR)	AUROC	accuracy	sensitivity	specificity
plasma RNA (n = 35)	Clinical (10%)	0.83	80.0%	90.9%	75.0%
	
	Clonal (10%)	0.67	71.4%	54.5%	79.2%
	
	Clonal optim. (5.75% )	0.75	82.9%	54.5%	95.8%

whole-blood DNA (n = 17)	Clinical (10%)	0.86	76.5%	100%	71.4%
	
	Clonal (10%)	0.69	70.6%	66.7%	71.4%
	
	Clonal optim. (5.75% )	0.76	82.3%	66.7%	85.7%

### Comparison of HIV-1 V3 RNA and DNA sequence population and their inferred coreceptor tropisms

The median intrapatient distance among HIV RNA or DNA sequences was, as expected, significantly smaller than the interpatient distance (see Additional files 1 and 2). However, the intrapatient variability with RNA (0.007) or with DNA (0.015) sequences was significantly lower than the intrapatient variability between RNA and DNA sequences (0.023), suggesting that the HIV-1 V3 DNA and RNA populations may contribute different information and complement each other in a patient. Phylogenetic analysis revealed that clusters of sequences with high support values (bootstrap >90%) corresponded to sequences drawn from the same patients, and there were no clusters composed of sequences from different patients. Clusters were preferentially (but not exclusively) composed of either paired RNA or DNA samples (phylogenetic tree is available as Additional file 1).

We next investigated whether a certain degree of intrapatient diversity between DNA and RNA populations results in a different co-receptor tropism prediction. When comparing geno2pheno clinical interpretation based on paired HIV-1 V3 DNA-RNA sequences obtained from 29 distinct patients, we observed 35/40 (87.5%) concordant predictions, 3/40 (7.5%) false positives, and 2/40 (5%) false negatives, using RNA-predicted X4-tropism as the reference outcome. The kappa statistics yielded a strength of agreement of 0.74 (95% CI: 0.53-0.95), with a Fisher's p-value < 0.0001. The AUROC obtained by predicting the geno2pheno RNA tropism from contemporary DNA sequences was 0.875.

### Viro-immunological follow up

Out of 28 patients with an R5-tropic virus by ESTA subsequently starting MVC, 22 had an available virological follow up at 12 weeks. All patients had a VL below 50 cp/ml, except two patients with 120 and 292 cp/ml. Virological follow-up at 24 weeks was available for 19 patients. All of these had a VL below 50 cp/ml, except two patients (different from those at three months) with a VL of 2,003 and 88 cp/ml.

Immunological follow-up at 12 weeks was available for 20 out of the 28 patients that started MVC. The median (IQR) CD4 increase was 84 (range -9 to 165) cells/mm3. A Wilcoxon test showed that this increase from baseline was statistically significant (p = 0.019). After 24 weeks of therapy, immunological follow-up was available for 19 patients. The median (IQR) CD4 increase was 46 (-9 to 143) cells/mm3(p = 0.053).

### Evolution of V3 sequences during MVC therapy

**Figure 3:**
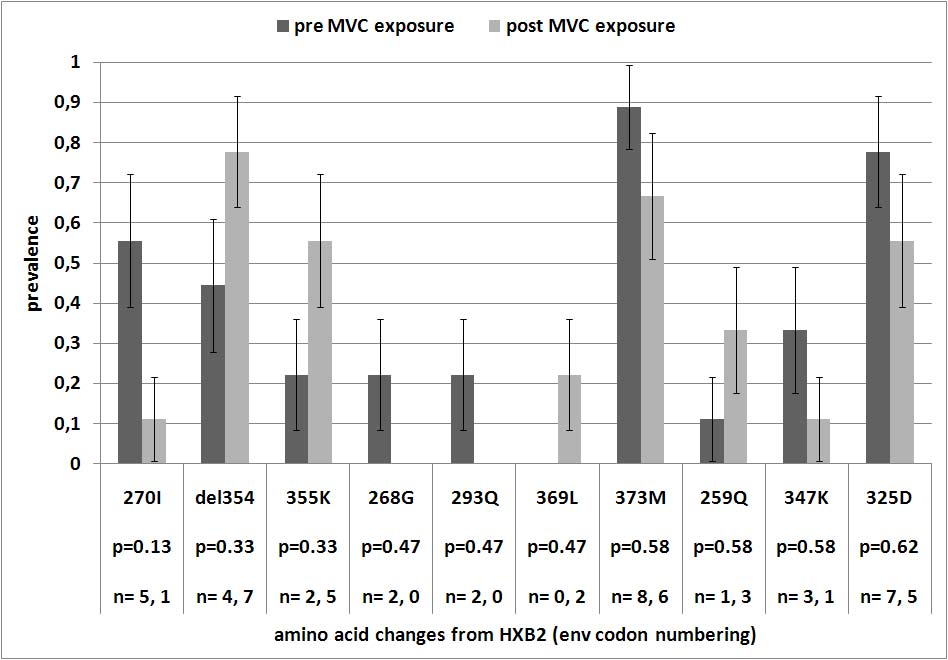
**Prevalence of mutations (with respect to HIV-1 HXB2) in the V3 loop from RNA samples, stratified by MVC exposure (n = 18, 9 sequences pre-MVC, 9 post-MVC). Mutations are shown in decreasing order by unadjusted p-value obtained from a Fisher's test comparing pre- vs**. post-MVC prevalence

Finally, we executed statistical tests for difference in proportions by looking at the whole set of mutations retrieved in the RNA samples with respect to HIV-1 HXB2 envelope reference, grouping sequences in pre- and post-MVC initiation (n = 18, 9 sequences pre-MVC, 9 post-MVC). Of the 9 pre-MVC sequences (all R5 by ESTA), 4 (44%) were classified as X4 by clinical geno2pheno interpretation, with FPR of 4.8%, 7.8%, 0.2%, and 0.8%. Tropism prediction did not change for any patient when considering post-MVC samples. No substitution was significantly associated with MVC exposure by correcting test statistics with Benjamini Hochberg (all p-values = 1) nor retaining raw unadjusted p-values; nonetheless a deletion at position 354, mutation 355K, 369L, and 259Q (all in the V3 loop) showed an increase in prevalence after MVC initiation. Figure 3 depicts mutations detected in the V3 loop in pre- and post-MVC samples with the lowest unadjusted p-values.

## Discussion

In the present study, viral tropism prediction by the HIV-1 gp120 V3-loop RNA genotype-based geno2pheno_[coreceptor] _interpretation in treatment-experienced subjects proved to be a valid alternative to ESTA tropism, yielding an AUROC of 0.83 using the clinical interpretation. The clonal interpretation showed a lower AUROC, although the difference with the clinical interpretation showed only a trend, probably due to the limited sample size. The geno2pheno_[coreceptor] _website suggests that only the clonal interpretation should be used for treatment-experienced patients, since the clinical system was trained only on treatment-naïve patients. However, our results show that the clinical interpretation is better (at least not inferior) than the clonal interpretation even in treatment-experienced patients. It is important to highlight the fact that the clinical interpretation needs contemporary VL, nadir CD4 and CD8 information in order to work properly. Interestingly, the clinical mode improved sensitivity much more than specificity with respect to the clonal mode. The sensitivity of V3 RNA genotyping followed by clonal geno2pheno_[coreceptor] _interpretation has been recently shown to increase by testing multiple aliquots of the plasma RNA extract [39], likely as a consequence of stochastic fluctuations of a minority X4 populations. Although repeated testing has not yet been established as a standard procedure, it is expected that its implementation would result in a higher concordance between tropism results obtained by V3 genotyping and the reference ESTA.

Another area under investigation is the optimal FPR to use with geno2pheno_[coreceptor] _as a cut-off for classification of R5 and X4-D/M viruses. Recent retrospective analysis of the MERIT trial data indicated that the most accurate FPR cutoff may be in the range from 2% to 5.75%, when triplicate PCR testing and fully automated sequencing is used [30]. However, there is currently no consensus on which cut-off to use in clinical practice. As for any classification test aimed at orienting a clinical intervention, the trade-off between specificity and sensitivity must be taken into account. A higher cut-off such as the 10% FPR was originally proposed [17] and is still recommended by the expert panel developing and maintaining the geno2pheno_[coreceptor] _system (see the German recommendations [40]). Using a higher cut-off translates into a conservative attitude, i.e. a lower probability to treat with maraviroc patients who may not benefit from it, at the expense of a higher probability not to treat patients who may benefit from the drug. It remains to be established whether this lower cut-off can be clinically more convenient in the genotypic screening of the general HIV patient population candidate to treatment with maraviroc.

One major advantage with genotyping is that even patients with non-reportable ESTA can be given prediction of tropism. By definition, ESTA is subject to a larger proportion of failures with respect to genotype due to virus polymorphisms invalidating the cloning procedure and an inherently lower rate of reverse transcription of a far larger virus genome region. Moreover, our efficiency of RNA genotyping at VL between 50 and 500 cp/ml (where ESTA is not even attempted) was 88%, whilst that of DNA genotyping was 100% at these viral loads.

Interestingly, in-silico tropism prediction using whole-blood DNA genotyping may be a solution when considering treatment switch to a CCR5-antagonists for patients with undetectable viral load. The perspective for treatment switches in these patients may be attractive, because of the good tolerability of MVC and because patients that are not at a late stage of disease are more likely to harbour a CCR5- rather than a CXCR4-tropic virus [4]. In these cases ESTA cannot be used, and RNA genotyping is often not sufficiently efficient. The performance of DNA-based clinical geno2pheno in predicting the ESTA result was comparable to that of RNA-based clinical geno2pheno (AUROC = 0.86). Thus, the use of V3 DNA sequence data for predicting co-receptor tropism definitely warrants further investigation as an appealing alternative to RNA.

As expected, we found some differences when comparing paired DNA and RNA sequences, consistent with the notion that the archived population may not correspond to the most prevalent virus in plasma, whose source are the productively infected cells. However, when comparing DNA and RNA tropism prediction by looking at contemporary samples, the degree of agreement was good, implying that such minor differences may not commonly translate into inappropriate indications. It remains to be established whether an X4 virus population detected in PBMC DNA in the context of R5 virus in plasma RNA can impair response to maraviroc. Although reported on a limited number of cases, this did not appear to be the case in the French GenoTropism study [41,42].

## Conclusion

HIV-1 tropism determination via plasma viral V3 RNA genotyping coupled with geno2pheno interpretation may represent a valid alternative to ESTA. The clinical validation of genotypic determination of viral tropism has been recently performed using retrospective samples from the MOTIVATE study [40] as well as in the GenoTropism study where the genotypic tropism test was able to predict response to maraviroc even in the group of patients with an R5 virus population as detected by standard Trofile^® ^[41,42]. As shown here, RNA tropism genotyping carries the advantage of a higher efficiency of tropism determination in patients with low copy number detectable viral loads. In addition to that, in perspective, DNA-based tropism prediction could be used in patients with undetectable VL who are candidates for treatment simplification/switch to a CCR5-antagonist. This option can support a more effective use of this class of agents at earlier stages when the probability of harbouring an R5 virus population is maximal. However, further investigations to unveil the evolutionary relationships between DNA and RNA populations are advisable before DNA genotyping can be indicated in clinical practice. In this context, ultra-deep sequencing studies may be appropriate to dissect the dynamics and role of DNA and RNA minority variants [43,44]. Most importantly, clinical validation of the use of HIV-1 genotyping, particularly with proviral DNA, for tropism assignment is also required before its widespread implementation.

## Competing interests

Maurizio Zazzi has received recent research funding from Pfizer; served as a consultant for Abbott Molecular, Boehringer Ingelheim, Gilead Sciences, and Janssen; and served on speakers' bureaus for Abbott, Bristol-Myers Squibb, Merck, and Pfizer.

Andrea De Luca received speakers honoraria, served as consultant or participated in advisory boards for GlaxoSmithKline, Gilead, Bristol-Myers Squibb, Abbott Virology, Tibotec-Janssen, Siemens Diagnostics and Monogram Biosciences.

Roberto Cauda has attended advisory boards or has been a consultant for Glaxo-SmithKline, Gilead, Bristol-Myers Squibb, Boehringer Ingelheim, Abbott Virology, Novartis, Pfizer, Schering-plough, and Merck Sharp & Dohme.

All other authors declare no competing interests.

## Authors' contributions

MCFP assisted with manuscript writing and statistical analyses; LB, MF, SDG and MC assisted with patients' care and data acquisition; FR, GM and AM assisted with laboratory assays; RC, MZ and ADL assisted with manuscript revision and research group leading. All authors read and approved the final manuscript.

## Supplementary Material

Additional file 1 **Evolutionary relationships of 155 V3 sequences obtained from 51 patients at different time points joining RNA and DNA samples + 1 outgroup (HIV-1 group J, V3 loop)**. Sequences are labelled by DNA/RNA type, by sampling date (the number before the type), and by patient's identifier (first one or two numbers). When considering all the 155 viral DNA/RNA V3 sequences obtained from all the 55 patients, the median (IQR) distance among all samples was 0.126 (0.101-0.159). The median (IQR) interpatient RNA-RNA distance was 0.133 (0.110-0.166) (n = 2,946 pairs) and DNA-DNA (n = 2,874) distance was 0.121 (0.095-0.152). The median (IQR) intrapatient RNA-RNA (n = 56), DNA-DNA (n = 52) and paired RNA-DNA (n = 120) distances were 0.007 (0.000-0.017), 0.015 (0.007-0.031) and 0.023 (0.015-0.031), respectively, with a Kruskal's p-value < 0.0001. Click here for file

Additional file 2 **Detailed classifications of tropism by different geno2pheno modes and ESTA on our data sets**. Click here for file
