# Supplementary figures and images for: Comparative determination of HIV-1 co-receptor tropism by Enhanced Sensitivity Trofile, gp120 V3-loop RNA and DNA genotyping
Source: Retrovirology. 2010 Jun 30;7:56. doi: 10.1186/1742-4690-7-56 (PMC2907304; doi:10.1186/1742-4690-7-56)

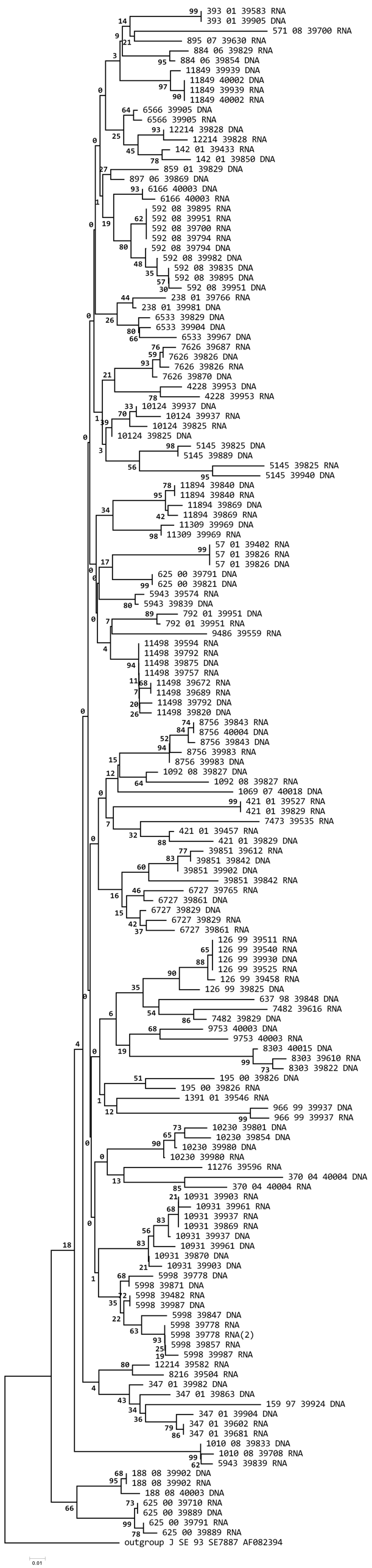

Supplement: Additional file 1 — Evolutionary relationships of 155 V3 sequences obtained from 51 patients at different time points joining RNA and DNA samples + 1 outgroup (HIV-1 group J, V3 loop). Sequences are labelled by DNA/RNA type, by sampling date (the number before the type), and by patient's identifier (first one or two numbers). When considering all the 155 viral DNA/RNA V3 sequences obtained from all the 55 patients, the median (IQR) distance among all samples was 0.126 (0.101-0.159). The median (IQR) interpatient RNA-RNA distance was 0.133 (0.110-0.166) (n = 2,946 pairs) and DNA-DNA (n = 2,874) distance was 0.121 (0.095-0.152). The median (IQR) intrapatient RNA-RNA (n = 56), DNA-DNA (n = 52) and paired RNA-DNA (n = 120) distances were 0.007 (0.000-0.017), 0.015 (0.007-0.031) and 0.023 (0.015-0.031), respectively, with a Kruskal's p-value < 0.0001. [file 1742-4690-7-56-S1.PNG]
