# Supplementary material for: Comparative determination of HIV-1 co-receptor tropism by Enhanced Sensitivity Trofile, gp120 V3-loop RNA and DNA genotyping
Source: Retrovirology. 2010 Jun 30;7:56. doi: 10.1186/1742-4690-7-56 (PMC2907304; doi:10.1186/1742-4690-7-56)
Supplement: Additional file 2 — Detailed classifications of tropism by different geno2pheno modes and ESTA on our data sets. [file 1742-4690-7-56-S2.DOCX]

**Additional file 2**. Detailed classifications of tropism by different geno2pheno modes and ESTA on our data sets.

| ESTA | ESTA_X4 | g2p_clonal_10_fpr | g2p_clinical_10_fpr | g2p_optim_5.75_fpr | sample_type |
| --- | --- | --- | --- | --- | --- |
| R5 | 0 | 0 | 0 | 0 | RNA |
| R5 | 0 | 0 | 0 | 0 | RNA |
| R5 | 0 | 0 | 0 | 0 | RNA |
| R5 | 0 | 0 | 0 | 0 | RNA |
| R5 | 0 | 0 | 1 | 0 | RNA |
| R5 | 0 | 0 | 0 | 0 | RNA |
| R5 | 0 | 0 | 0 | 0 | RNA |
| R5 | 0 | 0 | 0 | 0 | RNA |
| R5 | 0 | 0 | 0 | 0 | RNA |
| R5 | 0 | 0 | 1 | 0 | RNA |
| R5 | 0 | 0 | 0 | 0 | RNA |
| R5 | 0 | 1 | 0 | 0 | RNA |
| R5 | 0 | 0 | 0 | 0 | RNA |
| R5 | 0 | 1 | 1 | 0 | RNA |
| R5 | 0 | 1 | 1 | 1 | RNA |
| R5 | 0 | 0 | 0 | 0 | RNA |
| R5 | 0 | 0 | 0 | 0 | RNA |
| R5 | 0 | 1 | 0 | 0 | RNA |
| R5 | 0 | 0 | 0 | 0 | RNA |
| R5 | 0 | 1 | 0 | 0 | RNA |
| R5 | 0 | 0 | 1 | 0 | RNA |
| R5 | 0 | 0 | 1 | 0 | RNA |
| R5 | 0 | 0 | 0 | 0 | RNA |
| R5 | 0 | 0 | 0 | 0 | RNA |
| D/M | 1 | 1 | 1 | 1 | RNA |
| D/M | 1 | 0 | 1 | 0 | RNA |
| D/M | 1 | 1 | 1 | 1 | RNA |
| D/M | 1 | 1 | 1 | 1 | RNA |
| X4 | 1 | 0 | 1 | 0 | RNA |
| D/M | 1 | 0 | 1 | 0 | RNA |
| D/M | 1 | 1 | 1 | 1 | RNA |
| D/M | 1 | 1 | 0 | 1 | RNA |
| D/M | 1 | 0 | 1 | 0 | RNA |
| D/M | 1 | 1 | 1 | 1 | RNA |
| D/M | 1 | 0 | 1 | 0 | RNA |
| R5 | 0 | 0 | 0 | 0 | DNA |
| R5 | 0 | 1 | 1 | 1 | DNA |
| R5 | 0 | 1 | 0 | 0 | DNA |
| R5 | 0 | 0 | 0 | 0 | DNA |
| R5 | 0 | 0 | 0 | 0 | DNA |
| R5 | 0 | 0 | 0 | 0 | DNA |
| R5 | 0 | 0 | 0 | 0 | DNA |
| R5 | 0 | 0 | 0 | 0 | DNA |
| R5 | 0 | 1 | 1 | 0 | DNA |
| R5 | 0 | 0 | 0 | 0 | DNA |
| R5 | 0 | 1 | 1 | 1 | DNA |
| R5 | 0 | 0 | 1 | 0 | DNA |
| R5 | 0 | 0 | 0 | 0 | DNA |
| R5 | 0 | 0 | 0 | 0 | DNA |
| D/M | 1 | 1 | 1 | 1 | DNA |
| D/M | 1 | 1 | 1 | 1 | DNA |
| D/M | 1 | 0 | 1 | 0 | DNA |
